# Dietary Patterns During Weight Loss Maintenance vs. Weight Regain: A Secondary Analysis of the Look AHEAD Trial

**DOI:** 10.3390/nu18020327

**Published:** 2026-01-20

**Authors:** Mary Catherine Prater, Frank L. Greenway, Ursula White

**Affiliations:** Division of Clinical Science, Pennington Biomedical Researcher Center, Baton Rouge, LA 70808, USA; catherine.prater@pbrc.edu (M.C.P.); frank.greenway@pbrc.edu (F.L.G.)

**Keywords:** weight regain, dietary patterns, sex differences, weight loss maintenance, Look AHEAD (Action for Health in Diabetes) trial, obesity, DASH diet, principal components analysis

## Abstract

**Background/Objectives**: Limited information exists on how dietary patterns change in adults who experience weight regain vs. those who maintain weight loss after lifestyle interventions. **Methods**: Five hundred fifty-two adults (60 ± 1.0 years, 33.8 ± 0.4 kg/m^2^) with type 2 diabetes mellitus from the Look AHEAD Trial achieved ≥ 7% weight loss after Year 1, completed follow-up visits through Year 4, and provided three 134-item food frequency questionnaires. Weight “regain” (WLR) was defined as regaining ≥ 50% of the initial weight lost. Dietary patterns were determined using established DASH diet scores (scale: 0–9) and principal component analysis (PCA; higher scores = more adherent). Repeated measures linear mixed models assessed group and sex differences in dietary patterns. **Results**: Dietary patterns were similar between groups during weight loss (baseline to Year 1). WLR DASH scores decreased more from Year 1 to Year 4 compared to “maintain” (WLM) (WLR: Y1: 5.66 ± 0.14, Y4: 4.60 ± 0.14; WLM: Y1: 5.49 ± 0.13, Y4: 4.92 ± 0.13; difference-*p* < 0.01). Of the two PCA-derived dietary patterns, Pattern 1 (vegetable, fruit, and fish) decreased more in WLR (WLR: Y1: 0.12 ± 0.16, Y4: −0.14 ± 0.16, WLM: Y1: 0.06 ± 0.14, Y4: 0.25 ± 0.15; difference-*p* < 0.01), while Pattern 2 (low-fiber grains and high-fat animal proteins) increased in WLR (WLR: Y1: 0.40 ± 0.11, Y4: 0.61 ± 0.11, WLM: Y1: 0.34 ± 0.10, Y4: 0.21 ± 0.10, difference-*p* < 0.01). Sex differences showed that only WLR women and WLM men increased sweets from Y1 to Y4 (WLR women Y1: 0.26 ± 0.04, Y4: 0.41 ± 0.04; *p* < 0.01; WLM men: Y1: 0.23 ± 0.04, Y4: 0.38 ± 0.04; *p* < 0.01). **Conclusions**: These data demonstrate that differences in dietary patterns between WLR and WLM emerge after the initial weight loss intervention with some sex differences. This suggests that longer-term shifts in dietary patterns after lifestyle interventions may influence weight loss maintenance.

## 1. Introduction

In 2021, overweight and obesity were associated with an estimated 3.7 million deaths [1], and they are significant risk factors for many chronic diseases. Weight loss reduces the risk of obesity-related morbidity and mortality and improves metabolic disease risk, including type 2 diabetes; however, weight regain remains one of the greatest challenges to obesity management [2,3]. NHANES estimates that only about 1 out of every 6 adults who have ever had overweight or obesity have maintained a weight loss of at least 10% for one year [4]. This highlights that individuals can successfully lose weight and adiposity, but sustaining weight loss remains difficult and poses a continued public health challenge.

Understanding the behaviors of individuals who have successfully maintained weight loss is necessary to understand effective strategies for long term weight loss maintenance. Investigations of successful weight loss maintainers have identified multiple behaviors that may facilitate weight loss maintenance, including self-monitoring of weight, regular exercise, and limiting the intake of certain foods [5,6,7]. Diet is consistently observed as an important component of weight loss maintenance. While multiple dietary factors, including reduced fat intake, reduced carbohydrate rich foods, and increased fruits and vegetables, have been observed, there remains little consensus on the role of dietary patterns that support weight loss maintenance [8,9].

Dietary patterns refer to the types and quantities of foods habitually consumed rather than individual nutrients, and limited data suggest that dietary patterns can influence weight regain after successful weight loss. The existing data reporting dietary patterns are limited by follow-up assessments of only one year or less and are often focused on analyzing weight loss maintenance as opposed to weight regain behaviors. Furthermore, it is now evident that a “one-size-fits-all” approach to weight loss maintenance may not be appropriate. The National Weight Control Registry identified multiple subgroups of successful weight loss maintenance behaviors, with differing levels of difficulty experienced by individuals maintaining weight loss being reported [10]. These differences in experiences with weight maintenance, coupled with limited prospective analyses of dietary patterns during weight loss maintenance, highlight the need for detailed investigations of dietary patterns in successful weight loss “maintainers” vs. weight loss “regainers”.

Herein, we have examined the dietary patterns of participants from the Look Action for Health and Diabetes (Look AHEAD; NCT00017953) trial who completed a 1-year intensive lifestyle intervention (ILI) or the Diabetes Support and Education (DSE) intervention and experienced either weight regain or weight loss maintenance after 4 years. This analysis also explored potential sex differences in the dietary patterns of weight loss maintenance. Our data show that the dietary patterns of the weight loss maintainers were characterized by diets rich in fruits and vegetables, while weight loss regainers showed a shift in dietary patterns towards increased energy from fats and a decreased fruit and vegetable intake during the “weight maintenance” phase. These findings provide compelling evidence that regainers shifted away from “healthy” dietary patterns after the initial weight loss intervention and suggest that approaches to reinforce “healthy” dietary patterns, including diets rich in fruits and vegetables and low in energy from fats, during the weight maintenance period are important to promote longer-term weight loss maintenance after lifestyle interventions. These data confirm existing assumptions of the importance of “healthy” dietary patterns in the context of weight loss maintenance through the analysis of prospective weight loss and weight regain periods, an approach not commonly available in the current literature.

## 2. Materials and Methods

### 2.1. Subject Characteristics

The study design and protocol from the Look AHEAD trial have been previously described in detail [11,12]. Briefly, Look AHEAD was a 16-site multicenter, randomized, parallel, controlled clinical trial designed to assess the effects of an intensive lifestyle intervention (ILI) to promote weight loss compared with Diabetes Support and Education (DSE; standard care) on CVD risk in individuals with a BMI > 25 kg/m^2^ (or >27 kg/m^2^ if taking insulin) and a type 2 diabetes diagnosis. In the first year, the ILI targeted ~7% weight loss through group support sessions, caloric restriction, recommended meal replacement use, and increased physical activity. After one year, the three-year maintenance phase included monthly group meetings and recommendations to use a single meal replacement per day and continue physical activity. The Diabetes Support and Education (DSE) group served as the active control group, receiving general information on dietary intake and physical activity through three group sessions per year, but no specific counseling targeting specific behavior modifications.

The present analyses were conducted using data from a subset of participants (*n* = 552) from the Look AHEAD trial (NCT00017953; participants from the Southwest Native American sites are not included) who successfully lost at least 7% of their initial body weight, provided three completed food frequency questionnaires (FFQ), and had follow-up data through the end of Year 4. Weight was collected annually in-person from participants. Participants were stratified into weight trajectory groups based on the magnitude of weight regained at Year 4 relative to the magnitude of weight lost by Year 1. “Weight loss regain” (WLR) was defined as regaining at least 50% of the initial weight lost ([Year 1 − baseline/Year 4 − Year 1] × 100) [13,14]. “Weight loss maintenance” (WLM) described participants who did not meet or exceed 50% weight regain at Year 4. The analyses performed herein were not conducted at the Look AHEAD Data Coordinating Center. This does not represent the work of the Look AHEAD study group.

### 2.2. Dietary and Physical Activity Assessments

Dietary intake was assessed in the first 50% of participants enrolled at each site [11,12]. Briefly, a semi-quantitative, 134-item food frequency questionnaire (FFQ) was administered at baseline, Year 1, and Year 4 to assess usual dietary intake over the previous six months. This FFQ was modified from the Diabetes Prevention Program (DPP) study protocol to include meal replacements. A certified interviewer provided instructions for completing FFQs and reviewed completed questionnaires for errors and completeness, while final quality control checks and analyses were completed by the Look AHEAD Diet Assessment Center (DAC), located at the University of South Carolina, Columbia, SC. The National Cancer Institute Health Habits and History Questionnaire HHHQ/DietSys program (version 3.0, 1993, National Cancer Institute, Rockville, MD, USA), including Look AHEAD specific programming, was used to estimate daily food group, energy, and nutrient intakes from the FFQs. Nutrient values and energy content estimates were obtained from the Nutrition Data System (NDS-R) Nutrition Coordinating Center, University of Minnesota, Minneapolis, MN (version 4.01_30, 1999). Food group servings were based upon the Food Guide Pyramid [15], and meal replacements were coded as dairy (beverage) and protein foods (food bars).

Self-reported physical activity was collected at 8 of the 16 sites using the Paffenbarger questionnaire at baseline, Year 1, and Year 4 [16], which provided an estimated weekly energy expenditure from moderate intensity physical activity.

### 2.3. Statistical Analysis

All analyses were conducted using SAS v9.4 (Cary, NC, USA) or R Statistical Software v4.5.0 (Vienna, Austria), using an alpha level of <0.05 for significance. Chi-squared tests and one-way ANOVAs were used to assess baseline characteristics of weight trajectory groups by sex. DASH Index dietary pattern scores, as described by Mellen and colleagues [17], were calculated using the R package (dietaryindex [18]). Percent weight change from baseline, self-reported nutrients, food groups, physical activity, and dietary pattern scores were all assessed using repeated measures linear mixed models (proc mixed) including factors of time, weight trajectory group (referred to as group), and sex. All data in these analyses were collected at baseline, Year 1, and Year 4. These models were all adjusted for covariates of randomization site, baseline age, randomization arm, and self-reported race. The Tukey–Kramer post hoc test was used to investigate significant time by group, and time by group by sex interactions. Data are presented as least squares means ± standard error unless otherwise noted.

Principal component analysis (PCA) was used to determine data driven dietary patterns using 23 food subgroups developed by the DAC (conducted in SAS using proc princomp). The Kaiser–Meyer–Olkin (KMO) Test and Bartlett’s Test of Sphericity were used to confirm the suitability of the food subgroup data for this analysis. Components (i.e., diet patterns) were retained based on inspection of scree plots, component interpretability, and eigenvalues. Factors were interpreted as loading onto the component if loadings were greater than |0.2|, a cutoff that has been used commonly in dietary pattern analysis applications [19], and the highest loadings were used to characterized the respective dietary patterns. First, PCA was conducted at baseline to describe the predominant dietary patterns in the population regardless of weight trajectory group. Second, the linear equations derived from the patterns retained at baseline were applied to follow-up data at Year 1 and Year 4, developing scores to evaluate how “adherence” to the baseline dietary patterns may have changed over time. PCA scores over time were assessed using the same repeated linear mixed models as described above.

## 3. Results

### 3.1. Participants and Baseline Characteristics

Baseline characteristics by weight trajectory and sex are presented in Table 1. On average, the participants included in the analysis were men and women with obesity (35.9 ± 5.89 kg/m^2^, and 58 ± 7 years), with no significant differences in BMI between groups, but differences in age (*p* < 0.001) driven by WLR women being younger than WLM men. There were also differences in education, race representation, and reported family incomes. A greater number of women had <13 years of education and were from minority (African American/Black or Other/Mixed) groups, while a lower proportion were from high income households. There were no differences in baseline HbA1c or lifestyle intervention assignments between groups.

### 3.2. Weight Trajectory

**Figure 1:**
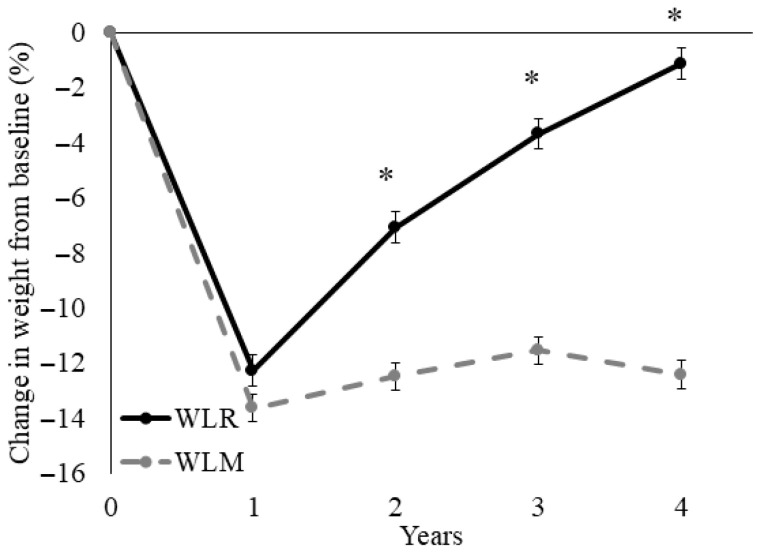
Time course for percent weight loss relative to baseline of a subset of participants from the Look AHEAD trial that successfully lost 7% of initial weight by Year 1. Data presented as least squares means ± standard error. Data were analyzed using repeated measures linear mixed models with main effects of time, group, and sex, with covariates of randomization site, age, randomization arm, and self-reported race. When significant time by group or time by group by sex interaction was detected, a Tukey–Kramer post hoc test was conducted. * indicates difference in WLR vs. WLM (*p* > 0.05). WLR—weight loss regain group, defined as participants that regained at least 50% of Year 1 weight loss by Year 4; WLM—weight loss maintenance group, defined as participants that did not regain at least 50% of Year 1 weight loss by Year 4.

The average percent change in weight from baseline to Year 4 for WLR (*n* = 250) and WLM (*n* = 302) is shown in Figure 1. Both groups lost similar amounts of weight by Year 1 (−12.3 ± 0.6% vs. −13.6 ± 0.5%; *p* = 0.08). The WLR (regain) group experienced relative increases in body weight compared to the WLM (maintain) group at Years 2–4 (all *p* < 0.01). There were no sex differences in weight trajectories between groups (*p* = 0.84).

### 3.3. Dietary Intake and Physical Activity

The estimated daily dietary intake and physical activity (PA) are presented in Table 2. Macronutrients were assessed as a percentage of energy intake. The percent energy intake from total fat and saturated fat decreased from baseline to Year 1 in both groups, with a greater increase from Year 1 to Year 4 in WLR vs. WLM (both *p* < 0.01). Conversely, the percent carbohydrate intake increased more in WLR from baseline to Year 1 (*p* = 0.005) as compared to WLM, and decreased more during the follow-up from Year 1 to Year 4 (*p* < 0.001). There were no differences between groups in percent protein intake and no sex differences for any macronutrients (*p* > 0.05). There were no differences between weight trajectory groups over time for self-reported total energy intake (EI) or PA (*p* > 0.10). There were overall effects of sex, with females consuming less energy (*p* < 0.01) and engaging in less PA (*p* < 0.01) than males, but there were no sex differences between groups for either EI (*p* = 0.84) or PA (*p* = 0.33).

### 3.4. Food Groups

From the FFQ responses, six broad food groups were developed at the DAC based on the Dietary Guidelines for Americans 2000 and the Food Guide Pyramid [15], including breads/grains, vegetables, fruits, protein foods, dairy foods, and fats and sweets. Sub-groupings of foods within these six main food groups have also been developed by the DAC and are available in the public dataset. Food groups are presented as servings per day per 1000 kcal in Table 3.

Overall, WLR was characterized by greater decreases in fruit (Year 1 to Year 4 *p* < 0.01) and dairy (Year 1 to Year 4 *p* < 0.01) consumption compared to WLM. The overall effect may be driven by decreases in fruits other than citrus in WLR compared to WLM (*p* < 0.01). However, the consumption of high-fat dairy increased more in WLR compared to WLM from Year 1 to Year 4 (*p* < 0.01), in contrast to the decreased overall dairy consumption in WLR that was observed. It is important to note that the WLR group consumed less dairy overall relative to the WLM group, but what they did consume was higher fat. Further, WLR reported greater decreases in dark green/deep yellow (*p* = 0.04) and cruciferous vegetables (*p* = 0.01) compared to WLM regardless of sex. This effect was not observed in overall vegetable intake (*p* = 0.27). Women reported a greater overall fruit, vegetable, dairy, and bread/grain consumption across all timepoints (main effect of sex *p* < 0.01) with no sex interactions. There were no differences in breads/grains reported by groups and no other effects for fruit, vegetable, bread/grain, or dairy food groups.

Sex differences between the weight trajectory groups showed that only WLR men increased their intake of overall protein foods and specifically high-fat fish intake from Year 1 to Year 4 (both *p* < 0.01). Furthermore, women in the WLR and WLM groups reported decreasing both protein foods and high-fat fish during weight loss (both *p* < 0.05), with no change from Year 1 to Year 4 (both *p* > 0.05). Regardless of sex, WLR increased high-fat poultry (*p* < 0.01) and egg (*p* = 0.03) consumption from Year 1 to Year 4 compared to WLM. Conversely WLM reported greater decreases in low-fat meat intake from baseline to Year 4 compared to WLR (*p* = 0.01). No other differences in protein foods were observed.

Sex and group differences in fat, oil, and sweets intake showed that all groups except for WLR men increased their intake from Year 1 to Year 4 (all *p* < 0.05). In the sweets and desserts subgroup specifically, only WLR women and WLM men increased their intake from Year 1 to Year 4 (both *p* < 0.01). Additionally, there were greater increases from baseline to Year 1 (*p* < 0.01) and greater decreases from Year 1 to Year 4 (*p* < 0.01) in meal replacement use in the WLR vs. the WLM group. A greater meal replacement usage was reported in women compared to men (main effect of sex; *p* < 0.01).

### 3.5. Dietary Patterns

#### 3.5.1. DASH Dietary Pattern

**Figure 2:**
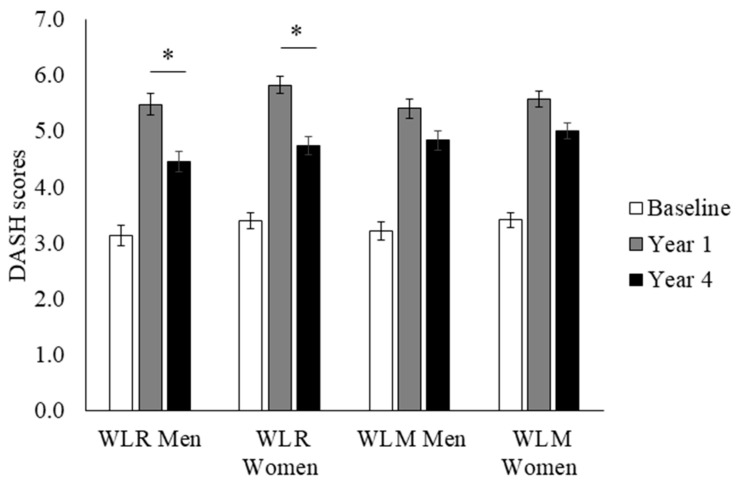
DASH index scores over time of a subset of participants from the Look AHEAD trial that successfully lost 7% of initial weight by Year 1. Data presented as least squares means ± standard error. Data were analyzed using repeated measures linear mixed models with main effects of time, group, and sex, with covariates of randomization site, age, randomization arm, and self-reported race. When significant time by group or time by group by sex interaction was detected, a Tukey–Kramer post hoc test was conducted. * indicates difference in WLR vs. WLM (*p* > 0.05). WLR—weight loss regain group, defined as participants that regained at least 50% of Year 1 weight loss by Year 4; WLM—weight loss maintenance group, defined as participants that did not regain at least 50% of Year 1 weight loss by Year 4.

The Dietary Approaches to Stop Hypertension (DASH) diet is a well characterized dietary pattern that emphasizes the consumption of fruits, vegetables, low-fat dairy products, reduced saturated and total fat, and reduced sodium intake. The DASH diet has been shown to support cardiometabolic health, and the DASH index, developed by Mellen and colleagues [17], leverages established nutrient goals in the assessment of individual adherence to the DASH dietary pattern. DASH scores range from 0 to 9, with scores ≥ 4.5 indicating a relative adherence to a DASH dietary pattern. Higher scores indicate better adherence. DASH scores and sub-scores in the WLR and WLM groups are presented in Figure 2. There was a greater decrease in DASH index scores from Year 1 to Year 4 in WLR vs. WLM (*p* < 0.01). Women had higher DASH scores regardless of group or time point compared to men (main effect of sex; *p* < 0.01); however, there were no sex differences by weight trajectory groups.

#### 3.5.2. Observed Dietary Patterns

**Figure 3:**
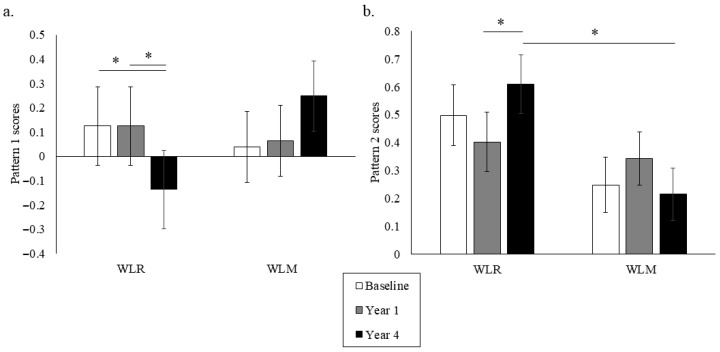
Data-driven dietary Patterns 1 (**a**) and 2 (**b**) over time of a subset of participants from the Look AHEAD trial that successfully lost 7% of initial weight by Year 1. Data presented as least squares means ± standard error. Data were analyzed using repeated measures linear mixed models with main effects of time, group, and sex, with covariates of randomization site, age, randomization arm, and self-reported race. When significant time by group or time by group by sex interaction was detected, a Tukey–Kramer post hoc test was conducted. * indicates difference in WLR vs. WLM (*p* > 0.05). WLR—weight loss regain group, defined as participants that regained at least 50% of Year 1 weight loss by Year 4; WLM—weight loss maintenance group, defined as participants that did not regain at least 50% of Year 1 weight loss by Year 4.

Principal component analysis (PCA) was used to describe the predominant dietary patterns observed at baseline regardless of weight trajectory group (Table 4). The first pattern tended to capture a greater vegetable intake, fruit intake, fish intake, and avoidance of high-fat meat. Pattern scores 1 and 2 were derived from the baseline PCA dietary patterns and calculated for each weight trajectory group to assess group differences in observed dietary patterns over time (Figure 3). WLR had a greater decrease in Pattern 1 scores compared to WLM from Year 1 to Year 4 (*p* < 0.01). WLR had a greater increase in Pattern 2 scores from Year 1 to Year 4 compared to WLM. Furthermore, WLR had higher Pattern 2 scores at Year 4 than WLM (*p* < 0.01). There were no sex differences for either pattern.

## 4. Discussion

Herein, we present novel findings demonstrating how dietary patterns change in adults who experience weight regain (WLR) vs. those who maintain weight loss (WLM) after 1 year in the Look AHEAD trial. These data highlight the differences in dietary patterns that primarily emerged in the follow-up (weight maintenance or regain) period rather than at baseline or during the weight loss phase. WLM was characterized by maintaining higher intakes of fruits, specific vegetables, and dairy, while WLR was related to a greater fat intake, including high-fat animal products. These results were supported by various dietary pattern scores, with WLM maintaining higher DASH scores and the “healthy” Pattern 1 scores by Year 4, while WLR showed a shift towards the identified Pattern 2 scores characterized by high-fat foods. Overall, these data suggest that shifts in dietary behaviors during the longer-term (4-year) follow-up phase can potentially influence weight loss maintenance success.

Our macronutrient results align with previous findings that a higher dietary fat intake is associated with obesity and weight regain [20,21]. Both analyses of individual food groups and the PCA dietary Pattern 2 suggest that shifting to a dietary pattern of greater fat intake through animal products may be influencing weight regain in this sample. Of note, a higher protein intake has been associated with weight maintenance [7,22], yet we did not observe any differences between WLR and WLM in the percent energy from protein intake. Weight loss has been shown to result in a decrease in anorexigenic hormones and an increase in orexigenic hormones that may drive food-seeking behaviors and physiologically undermine weight loss maintenance efforts [23]. Dietary fat is believed to increase the palatability of foods while also increasing the energy density. Some evidence suggests that dietary fat may drive appetite response, leading to a higher EI, especially in free-living conditions (reviewed by Benelam [24]). While we did not capture differences in total EI in this sample, the greater reported intake of high-fat foods may be promoting energy imbalances and weight regain through these appetitive mechanisms.

Fruit and vegetable intake was clearly associated with successful weight loss maintenance, as characterized by the analysis of individual food groups, including the DASH dietary pattern score and PCA-derived dietary patterns. More specifically, we found similar reported increases in the consumption of fruits and vegetables during weight loss regardless of the weight trajectory group, but the WLR group reported a reduced fruit and vegetable intake during follow-up relative to the WLM group. Dietary patterns with a high fruit and vegetable content, such as DASH, have been repeatedly shown to support weight loss maintenance and improve cardiometabolic health [20,22,25,26,27,28,29], suggesting that maintaining “healthy” dietary patterns that are high in fruit and vegetable intake may support weight loss maintenance. Fruit and vegetable intake may be supporting weight loss maintenance through an increased fiber intake and decreased energy density of foods, leading to a decreased EI while maintaining food volume [30]. Interestingly, maintaining a similar level of diet quality up to three years after the initial weight loss intervention was characteristic of successful weight loss maintenance, thus implicating overall dietary quality as a potential intervention target for promoting weight loss maintenance.

Sex-specific effects in dietary patterns were observed in this analysis and may offer important information for improving weight maintenance strategies. In addition to women tending to have overall higher DASH adherence scores regardless of weight trajectory group, increases in sweets consumption were observed in WLR women and WLM men. Conversely, WLR men reported greater increases in protein foods and high-fat fish. In total, these data suggest that different shifts in dietary behavior may be driving weight regain between sexes. Sex differences in dietary patterns may relate to sex differences in food cravings and the processing of food cues. Reports of sex differences in food cravings align with our observations, suggesting that women tend to experience cravings for sweets while men tend to crave savory foods (reviewed in [31]). Though inconsistent regarding sweet foods specifically, men and women have also been found to have differences in neural activation in response to visual and gustatory food stimuli [32]. These data suggest that men and women process food cues differently, which may drive sex differences in cravings, appetite, and dietary behavior [33]. Though speculative, these differences in neural responses to food cues may provide some explanation as to why increases in reporting sweets and desserts in dietary patterns may be associated with weight regain in women but weight maintenance in men, yet a shift towards protein and high-fat foods may be characteristic of male weight loss regain patterns. Regardless of sex, increases in sweets and high-fat meats have been associated with obesity and weight regain [28,34]. A further investigation of sex differences in dietary behavior may be warranted to best inform tailored weight loss maintenance approaches.

Broadly, the dietary patterns observed in this analysis provide evidence to support the strategy of consuming low-energy-density foods to promote weight maintenance. Energy density refers to the amount of energy per gram of a food, and energy density can be reduced by selecting foods with a higher water and fiber content such as fruits, vegetables, and whole grains. Reducing the energy density of the diet as a method to promote reduced energy intake has been promoted by the Dietary Guidelines for Americans since as early as 2005 [35]. To demonstrate the effect of food volume on appetite, the provision of a low-energy-density first course (“preload”) has been shown to decrease ad libitum energy intake during a meal and promote satiation in a controlled setting [36]. Furthermore, outpatient weight loss trials incorporating low-energy-density foods as part of the weight loss program observed greater weight loss compared to high-energy-density foods, likely due to the lower energy intake at self-selected meals [37,38,39]. Across the analyses presented, dietary patterns rich in low-energy-density foods, such as fruits and vegetables, are consistently reported by those who were more successful in weight loss maintenance. Conversely, the dietary patterns present in weight regain are characterized by a high fat content. Ultimately, the consumption of low-energy-density diets was characteristic of weight loss maintenance in this analysis and provides evidence supporting the adherence to low-energy-density diets in general as a strategy for supporting weight loss maintenance.

This analysis supports the role of high-quality dietary patterns and low energy density as characteristic of participants who avoid weight regain. However, it is important to acknowledge the challenges of behavior change. While it is possible to help an individual develop new habits and behaviors through methods including cognitive behavioral therapy, the effectiveness of these approaches in weight maintenance may have limitations [40,41]. Biological predispositions related to taste preference and food reward—processes that may be genetically determined [42]—may increase resistance to sustained dietary behavior change aligned with generalized recommendations. Accordingly, the divergent dietary patterns observed among adults who experience weight regain may in part reflect these underlying biological constraints rather than a simple non-compliance with dietary advice. With this in mind, it may be necessary to develop more tailored dietary plans for weight maintenance that consider taste preferences while maintaining energy balance.

This analysis has many strengths. The high quality of data collection and standardized data analysis at a centralized DAC support the strength of the self-reported data presented in this analysis. Furthermore, this analysis had several methodological strengths, including repeated measures of dietary intake over four years, the suitability of FFQs for capturing habitual diet, and the use of two complementary approaches for assessing dietary patterns (DASH scores and PCA). Though strong methods for assessing dietary patterns were leveraged, the use of self-reported measures to assess EI and PA, which are inherently subject to recall bias [43], may have limited our ability to draw specific conclusions regarding energy balance. Specifically, it is important to note that FFQs are limited in their ability to capture EI but are very well suited to assess dietary patterns and usual food choices. In addition, obesity and the physiology of weight loss maintenance are complex and multifactorial processes. This analysis focused on dietary intake behaviors as an influential factor in weight loss maintenance, though there are many other physiological and psychological factors that warrant further exploration. Finally, this analysis includes individuals with both overweight/obesity and type 2 diabetes from the Look AHEAD trial. While these dietary patterns may not be generalizable to younger populations or those free from type 2 diabetes, these data provide a foundation for potential dietary approaches that support weight maintenance that can be applied in future studies.

## 5. Conclusions

In conclusion, we have shown that there are measurable changes in dietary patterns between WLR and WLM following weight loss from a 1-year behavioral intervention, and these changes occur primarily after the initial weight loss phase. WLM was characterized by successfully maintaining multiple dietary behaviors that improved diet quality up to three years after the weight loss intervention, while, conversely, WLR shifted key dietary behaviors related to dietary fat and fruit and vegetable intake that may have influenced weight regain. Sex differences were observed in some dietary behaviors, likely warranting a further investigation of how sex-specific behaviors can influence weight trajectory. While these findings confirm some existing approaches for weight loss maintenance, this analysis is one of few large prospective assessments of dietary choices across both weight loss and weight loss maintenance. This work provides a firm rationale for prospective investigations of dietary approaches to support weight loss maintenance that can inform tailored dietary recommendations.

## Figures and Tables

**Table 1:** Baseline characteristics by weight trajectory and sex.

	WLR		WLM		*p* Values
	Men	Women	Men	Women	
Sex (*n* (%))	102 (41)	148 (59)	120 (40)	182 (60)	0.85
Education (*n* (%))					<0.01
<13 years or missing	12 (12)	37 (25)	11 (9)	58 (32)	
13–16 years	33 (32)	62 (42)	37 (31)	59 (32)	
>16 years	57 (56)	49 (33)	72 (60)	65 (36)	
Race (*n* (%))					<0.01
African American/Black	8 (8)	25 (17)	9 (8)	30 (16)	
Other/Mixed	5 (5)	15 (10)	11 (9)	40 (22)	
White	89 (87)	108 (73)	100 (83)	112 (62)	
Income (*n* (%))					<0.01
<USD 20 K or missing	12 (12)	19 (24)	36 (16)	39 (21)	
USD 20 K–40 K	10 (10)	8 (20)	30 (7)	46 (25)	
USD 40 K–60 K	13 (13)	20 (18)	26 (17)	38 (21)	
USD 60 K–80 K	19 (19)	19 (18)	27 (16)	25 (14)	
>USD 80 K	48 (47)	54 (20)	29 (45)	34 (19)	
Treatment arm (*n* (%))					0.62
Intensive lifestyle Intervention	96 (94)	134 (91)	108 (90)	163 (90)	
Diabetes Support and Education	6 (6)	14 (9)	12 (10)	19 (10)	
Age (years)	59.1 ± 7.31	56.4 ± 7.05	60.1 ± 6.7	57.9 ± 7.57	<0.01
BMI (kg/m^2^)	35.6 ± 5.72	36.3 ± 6.0	35.1 ± 5.46	36.4 ± 6.14	0.246
HbA1c (%)	7.21 ± 1.24	7.32 ± 1.15	7.19 ± 1.11	7.39 ± 1.18	0.45

Categorical data presented as counts (percent) and tested using chi-squared test. Continuous data presented as means ± SD and tested using a one-way ANOVA. BMI—body mass index; HbA1c—hemoglobin A1c; WLR—weight loss regain group, defined as participants that regained at least 50% of Year 1 weight loss by Year 4; WLM—weight loss maintenance group, defined as participants that did not regain at least 50% of Year 1 weight loss by Year 4.

**Table 2:** Estimated dietary intake and physical activity of men and women who experienced weight regain and weight maintenance in the Look AHEAD study.

	WLR						WLM						Time	Group	T × G	Sex	T × G × S
	Men (*n* = 102)			Women (*n* = 148)			Men (*n* = 120)			Women (*n* = 182)							
	Baseline	Y1	Y4	Baseline	Y1	Y4	Baseline	Y1	Y4	Baseline	Y1	Y4					
Estimated energy intake (kcals/day) ^1^	2170 ± 91	1810 ± 77	1854 ± 80	1801 ± 77	1455 ± 65	1498 ± 67	2175 ± 84	1774 ± 71	1722 ± 73	1827 ± 69	1448 ± 58	1446 ± 60	<0.01	0.50	0.17	<0.01	0.84
Total fat (% energy)	40.2 ± 0.8	32.9 ± 0.9	36.5 ± 0.8	41.1 ± 0.7	34.2 ± 0.8	37.7 ± 0.7	39.9 ± 0.8	33.9 ± 0.8	35.7 ± 0.8	40.6 ± 0.8	35.0 ± 0.7	36.5 ± 0.6	<0.01	0.75	<0.01	0.05	0.97
Saturated fat (% energy)	13.0 ± 0.4	9.6 ± 0.4	11.4 ± 0.4	13.4 ± 0.3	10.0 ± 0.3	12.0 ± 0.3	13.2 ± 0.3	10.2 ± 0.3	11.2 ± 0.3	13.0 ± 0.3	10.2 ± 0.3	11.0 ± 0.3	<0.01	0.60	<0.01	0.41	0.84
Carbohydrates (% energy)	43.1 ± 1.0	52.1 ± 1.1	46.7 ± 1.0	43.5 ± 0.8	51.7 ± 0.9	47.3 ± 0.8	43.8 ± 0.9	50.5 ± 1.0	48.8 ± 0.9	43.9 ± 0.7	50.2 ± 0.8	48.5 ± 0.7	<0.01	0.73	<0.01	0.99	0.67
Protein (% energy)	17.5 ± 0.3	18.0 ± 0.3	18.3 ± 0.3	17.1 ± 0.3	18.3 ± 0.3	17.9 ± 0.3	17.1 ± 0.3	18.1 ± 0.3	17.5 ± 0.3	16.9 ± 0.3	18.4 ± 0.3	18.1 ± 0.2	<0.01	0.37	0.18	0.81	0.10
Physical activity (kcal/week) ^#^	973 ± 255	2295 ± 320	1019 ± 299	501 ± 231	1255 ± 283	995 ± 266	1138 ± 226	2394 ± 282	1840 ± 263	757 ± 202	1604 ± 259	1343 ± 240	<0.01	0.04	0.23	<0.01	0.33

^1^ Least squares means ± Standard error. Data were analyzed using repeated measures linear mixed models with main effects of time, group, and sex, with covariates of randomization site, age, randomization arm, and self-reported race. ^#^ Physical activity: WLR men, *n* = 50; WLR women, *n* = 70; WLM men, *n* = 67; WLM women, *n* = 72. WLR—weight loss regain group, defined as participants that regained at least 50% of Year 1 weight loss by Year 4; WLM—weight loss maintenance group defined as participants that did not regain at least 50% of Year 1 weight loss by Year 4. T × G—time by group interaction; T × G × S—time by group by sex interaction.

**Table 3:** Estimated servings per 1000 kcals per day of Food Guide Pyramid food groups and subgroups in weight loss regain and weight loss maintain groups from the Look AHEAD study over the course of the intervention and follow-up periods.

	WLR						WLM						Time	Group	T × G	Sex	T × G × S
	Men (*n* = 102)			Women (*n* = 148)			Men (*n* = 120)			Women (*n* = 182)							
Food Groups and Subgroups ^1^	Baseline	Y1	Y4	Baseline	Y1	Y4	Baseline	Y1	Y4	Baseline	Y1	Y4					
***Bread/grains***	1.59 ± 0.06	1.23 ± 0.06	1.32 ± 0.06	1.74 ± 0.05	1.21 ± 0.05	1.43 ± 0.05	1.55 ± 0.05	1.20 ± 0.05	1.23 ± 0.05	1.89 ± 0.04	1.30 ± 0.04	1.46 ± 0.04	<0.01	0.58	0.21	<0.01	0.81
High-fiber/low-fat grains	0.41 ± 0.04	0.33 ± 0.04	0.37 ± 0.04	0.45 ± 0.04	0.35 ± 0.03	0.46 ± 0.04	0.43 ± 0.04	0.32 ± 0.04	0.39 ± 0.04	0.52 ± 0.03	0.43 ± 0.03	0.51 ± 0.03	<0.01	0.12	0.94	<0.01	0.55
Low-fiber/high-fat grains	0.28 ± 0.02	0.16 ± 0.02	0.22 ± 0.02	0.27 ± 0.02	0.16 ± 0.02	0.17 ± 0.01	0.23 ± 0.02	0.16 ± 0.02	0.16 ± 0.02	0.28 ± 0.02	0.16 ± 0.01	0.17 ± 0.01	<0.01	0.10	0.13	0.78	0.15
Low-fiber/low-fat grains	0.90 ± 0.05	0.73 ± 0.05	0.73 ± 0.05	1.02 ± 0.04	0.71 ± 0.04	0.80 ± 0.04	0.89 ± 0.05	0.73 ± 0.04	0.68 ± 0.04	1.09 ± 0.04	0.72 ± 0.04	0.78 ± 0.04	<0.01	0.98	0.33	<0.01	0.76
***Vegetables***	1.49 ± 0.10	2.16 ± 0.13	1.83 ± 0.12	1.84 ± 0.08	2.36 ± 0.11	2.05 ± 0.10	1.36 ± 0.09	1.94 ± 0.12	1.72 ± 0.11	1.80 ± 0.07	2.59 ± 0.10	2.26 ± 0.09	<0.01	0.87	0.27	<0.01	0.12
Tomatoes	0.32 ± 0.03	0.40 ± 0.04	0.36 ± 0.04	0.35 ± 0.03	0.42 ± 0.03	0.39 ± 0.03	0.31 ± 0.03	0.38 ± 0.04	0.33 ± 0.03	0.39 ± 0.02	0.51 ± 0.03	0.47 ± 0.03	<0.01	0.27	0.63	<0.01	0.39
Dark green/deep yellow	0.29 ± 0.03	0.42 ± 0.04	0.35 ± 0.04	0.35 ± 0.03	0.49 ± 0.03	0.42 ± 0.03	0.24 ± 0.03	0.36 ± 0.04	0.36 ± 0.03	0.34 ± 0.02	0.55 ± 0.03	0.48 ± 0.03	<0.01	0.94	0.04	<0.01	0.41
Cruciferous	0.20 ± 0.02	0.31 ± 0.03	0.25 ± 0.03	0.27 ± 0.02	0.34 ± 0.03	0.28 ± 0.03	0.16 ± 0.02	0.27 ± 0.03	0.27 ± 0.03	0.23 ± 0.02	0.37 ± 0.03	0.34 ± 0.02	<0.01	0.89	<0.01	<0.01	0.42
Other vegetables	0.68 ± 0.05	1.03 ± 0.07	0.86 ± 0.06	0.87 ± 0.04	1.11 ± 0.06	0.96 ± 0.05	0.66 ± 0.05	0.93 ± 0.06	0.75 ± 0.06	0.84 ± 0.04	1.16 ± 0.05	0.97 ± 0.05	<0.01	0.34	0.84	<0.01	0.18
***Fruits***	1.08 ± 0.09	1.56 ± 0.10	1.35 ± 0.10	1.32 ± 0.08	1.61 ± 0.08	1.47 ± 0.09	1.09 ± 0.08	1.43 ± 0.09	1.51 ± 0.09	1.29 ± 0.07	1.60 ± 0.07	1.66 ± 0.08	<0.01	0.57	<0.01	<0.01	0.45
Citrus	0.22 ± 0.03	0.27 ± 0.03	0.21 ± 0.04	0.29 ± 0.03	0.24 ± 0.03	0.25 ± 0.03	0.26 ± 0.03	0.30 ± 0.03	0.28 ± 0.03	0.27 ± 0.03	0.27 ± 0.02	0.30 ± 0.03	0.718	0.09	0.17	0.51	0.66
Other fruits	0.85 ± 0.08	1.30 ± 0.09	1.14 ± 0.09	1.03 ± 0.07	1.37 ± 0.07	1.22 ± 0.07	0.83 ± 0.07	1.13 ± 0.08	1.23 ± 0.08	1.02 ± 0.06	1.33 ± 0.06	1.36 ± 0.07	<0.01	0.99	<0.01	<0.01	0.60
***Dairy***	0.74 ± 0.08	1.51 ± 0.10	1.12 ± 0.10	0.99 ± 0.07	2.10 ± 0.08	1.59 ± 0.08	0.87 ± 0.07	1.61 ± 0.09	1.57 ± 0.09	0.94 ± 0.06	1.79 ± 0.07	1.55 ± 0.07	<0.01	0.35	<0.01	<0.01	0.10
High-fat diary	0.29 ± 0.03	0.16 ± 0.03	0.26 ± 0.03	0.38 ± 0.03	0.20 ± 0.02	0.32 ± 0.02	0.36 ± 0.03	0.23 ± 0.03	0.28 ± 0.03	0.34 ± 0.02	0.19 ± 0.02	0.23 ± 0.02	<0.01	0.86	0.02	0.32	0.78
Low-fat dairy	0.62 ± 0.07	0.49 ± 0.06	0.57 ± 0.07	0.73 ± 0.06	0.62 ± 0.05	0.81 ± 0.06	0.66 ± 0.06	0.61 ± 0.05	0.72 ± 0.06	0.71 ± 0.05	0.65 ± 0.04	0.84 ± 0.05	<0.01	0.12	0.32	<0.01	0.91
***Protein foods***	1.44 ± 0.06	1.22 ± 0.05	1.41 ± 0.06	1.54 ± 0.05	1.26 ± 0.05	1.38 ± 0.05	1.38 ± 0.05	1.25 ± 0.05	1.25 ± 0.05	1.47 ± 0.04	1.32 ± 0.04	1.42 ± 0.04	<0.01	0.46	0.01	0.01	0.04
High-fat fish	0.05 ± 0.00	0.02 ± 0.00	0.03 ± 0.00	0.03 ± 0.00	0.02 ± 0.00	0.03 ± 0.00	0.03 ± 0.00	0.02 ± 0.00	0.03 ± 0.00	0.04 ± 0.00	0.02 ± 0.00	0.02 ± 0.00	<0.01	0.11	0.02	0.03	<0.01
Low-fat fish	0.08 ± 0.01	0.12 ± 0.01	0.10 ± 0.01	0.09 ± 0.01	0.12 ± 0.01	0.10 ± 0.01	0.07 ± 0.01	0.10 ± 0.01	0.08 ± 0.01	0.08 ± 0.01	0.11 ± 0.01	0.11 ± 0.01	<0.01	0.30	0.33	0.27	0.55
High-*n*-3 fatty acid fish	0.06 ± 0.01	0.08 ± 0.01	0.06 ± 0.01	0.07 ± 0.01	0.07 ± 0.01	0.08 ± 0.01	0.05 ± 0.01	0.07 ± 0.01	0.06 ± 0.01	0.06 ± 0.01	0.07 ± 0.01	0.08 ± 0.01	<0.01	0.52	0.56	0.12	0.98
Dried beans	0.14 ± 0.02	0.16 ± 0.02	0.15 ± 0.02	0.16 ± 0.01	0.15 ± 0.02	0.15 ± 0.02	0.11 ± 0.02	0.14 ± 0.02	0.15 ± 0.02	0.16 ± 0.01	0.17 ± 0.01	0.17 ± 0.01	0.11	0.84	0.17	0.11	0.96
Eggs	0.14 ± 0.01	0.07 ± 0.01	0.11 ± 0.02	0.14 ± 0.01	0.08 ± 0.01	0.11 ± 0.01	0.12 ± 0.01	0.09 ± 0.01	0.10 ± 0.01	0.15 ± 0.01	0.11 ± 0.01	0.12 ± 0.01	<0.01	0.57	0.04	0.17	0.79
High-fat meat	0.40 ± 0.03	0.22 ± 0.02	0.29 ± 0.02	0.34 ± 0.02	0.18 ± 0.02	0.22 ± 0.02	0.35 ± 0.02	0.21 ± 0.02	0.23 ± 0.02	0.30 ± 0.02	0.18 ± 0.01	0.20 ± 0.02	<0.01	0.03	0.06	<0.01	0.57
Low-fat meat	0.14 ± 0.02	0.14 ± 0.02	0.17 ± 0.02	0.16 ± 0.02	0.12 ± 0.02	0.14 ± 0.02	0.17 ± 0.02	0.15 ± 0.02	0.15 ± 0.02	0.17 ± 0.01	0.14 ± 0.01	0.13 ± 0.01	0.02	0.58	0.02	0.36	0.43
High-fat poultry	0.04 ± 0.01	0.02 ± 0.00	0.04 ± 0.00	0.04 ± 0.00	0.02 ± 0.00	0.02 ± 0.00	0.04 ± 0.01	0.02 ± 0.00	0.02 ± 0.00	0.04 ± 0.00	0.02 ± 0.00	0.02 ± 0.00	<0.01	0.11	<0.01	0.02	0.41
Low-fat poultry	0.25 ± 0.02	0.28 ± 0.02	0.28 ± 0.02	0.30 ± 0.02	0.34 ± 0.02	0.31 ± 0.02	0.24 ± 0.02	0.24 ± 0.02	0.26 ± 0.02	0.30 ± 0.02	0.34 ± 0.02	0.34 ± 0.02	<0.01	0.95	0.77	<0.01	0.51
Nuts and seeds	0.12 ± 0.02	0.09 ± 0.02	0.14 ± 0.02	0.16 ± 0.02	0.10 ± 0.02	0.15 ± 0.02	0.16 ± 0.02	0.13 ± 0.02	0.13 ± 0.02	0.15 ± 0.02	0.12 ± 0.02	0.18 ± 0.02	<0.01	0.23	0.38	0.21	0.08
Soy products	0.02 ± 0.02	0.03 ± 0.02	0.04 ± 0.02	0.08 ± 0.02	0.08 ± 0.02	0.07 ± 0.02	0.04 ± 0.02	0.05 ± 0.02	0.05 ± 0.02	0.04 ± 0.02	0.06 ± 0.02	0.06 ± 0.02	0.43	0.83	0.85	0.06	0.42
***Fats, oils, and sweets***	0.88 ± 0.07	0.52 ± 0.06	0.60 ± 0.06	1.09 ± 0.06	0.62 ± 0.05	0.94 ± 0.05	0.94 ± 0.06	0.53 ± 0.06	0.75 ± 0.06	1.03 ± 0.05	0.62 ± 0.05	0.78 ± 0.05	<0.01	0.97	0.98	<0.01	0.01
Sweets and desserts	0.34 ± 0.05	0.24 ± 0.04	0.23 ± 0.04	0.47 ± 0.04	0.26 ± 0.04	0.41 ± 0.04	0.43 ± 0.04	0.23 ± 0.04	0.38 ± 0.04	0.41 ± 0.04	0.28 ± 0.03	0.36 ± 0.03	<0.01	0.37	0.59	0.03	<0.01
Fats and oils	0.55 ± 0.05	0.28 ± 0.04	0.37 ± 0.05	0.63 ± 0.04	0.37 ± 0.04	0.53 ± 0.04	0.51 ± 0.05	0.31 ± 0.04	0.38 ± 0.04	0.63 ± 0.04	0.34 ± 0.03	0.42 ± 0.03	<0.01	0.35	0.37	<0.01	0.15
Coffee and tea	0.83 ± 0.14	1.07 ± 0.15	0.87 ± 0.16	0.90 ± 0.12	1.06 ± 0.13	1.13 ± 0.13	0.91 ± 0.13	1.09 ± 0.14	1.06 ± 0.15	0.79 ± 0.11	0.97 ± 0.11	1.10 ± 0.12	<0.01	0.90	0.54	0.80	0.90
Meal replacements	0.02 ± 0.03	1.06 ± 0.09	0.49 ± 0.07	0.05 ± 0.02	1.46 ± 0.07	0.63 ± 0.06	0.03 ± 0.02	0.95 ± 0.08	0.75 ± 0.07	0.07 ± 0.02	1.13 ± 0.07	0.65 ± 0.06	<0.01	0.57	<0.01	<0.01	0.11

^1^ Least squares means ± standard error. Data presented as servings per 1000 kcals per day. Data were analyzed using repeated measures linear mixed models with main effects of time, group, and sex, with covariates of randomization site, age, randomization arm, and self-reported race. Main Food Guide Pyramid defined food groups shown in bold and italics. WLR—weight loss regain group, defined as participants that regained at least 50% of Year 1 weight loss by Year 4; WLM—weight loss maintenance group, defined as participants that did not regain at least 50% of Year 1 weight loss by Year 4. T × G—time by group interaction; T × G × S—time by group by sex interaction.

**Table 4:** ^1^ Data-derived dietary patterns were developed using PCA. Patterns are described by food groups with loadings > |0.20|. Patterns were retained based on inspection of scree plots, interpretability, and eigenvalues.

Pattern 1 ^1^	Pattern 2
Greater consumption of all vegetable subgroups, fruits other than citrus, high-fiber/low-fat grains, low-fat fish, and high-*n*-3 fish. Avoidance of high-fat meats and low-fiber/high-fat grains.	Greater consumption of low-fiber/high-fat grains, high-fat fish, high-fat poultry, low-fat poultry, and high-fat meat. Avoidance of low-fat dairy and sweets and desserts.

## Data Availability

Data from Look AHEAD: Action for Health in Diabetes (Look AHEAD) [(Version 9) https://doi.org/10.58020/wr3g-1218] reported here are available for request at the NIDDK Central Repository (NIDDK-CR) website, Resources for Research (R4R), https://repository.niddk.nih.gov/.

## Abbreviations

The following abbreviations are used in this manuscript:

ILI	Intensive Lifestyle Intervention
DSE	Diabetes Support and Education
FFQ	Food Frequency Questionnaires
WLR	Weight Loss Regain
WLM	Weight Loss Maintenance
DPP	Diabetes Prevention Program
PCA	Principal Component Analysis
KMO	Kaiser–Meyer–Olkin
DAC	Diet Assessment Center
PA	Physical Activity
EI	Energy Intake
DASH	Dietary Approaches to Stop Hypertension
